# Injury epidemiology and publishing injury research

**DOI:** 10.5249/jivr.v4i1.200

**Published:** 2012-01

**Authors:** Homayoun Sadeghi-Bazargani

**Affiliations:** ^a^Injury Epidemiology & Prevention Research Center, Statistics & Epidemiology Department, Tabriz University of medical Sciences, Tabriz, Iran

In the Dictionary of Epidemiology, Professor Last offers the following definition: “Epidemiology is the study of the distribution and determinants of health-related states or events in specified populations, and the application of this study to the control of health problems”.^1^ I similarly try to define injury epidemiology as the study of the distribution and determinants of injuries and safety related states-events in specified populations, and the application of this study to prevent injuries and promote safety.

The published research on injuries falls into this category only if it is produced based on the general perspectives of epidemiology and meets the necessary methodological standards. Injury epidemiology needs to be redirected from the largely descriptive studies, towards applying rigorous analytical methods for defining the underlying casual patterns of injury, determinants of the injury incidence, severity, and outcomes; and designing, implementing and rigorously evaluating interventions.^2^Although many journals try to be quite rigorous in assessing the methodological soundness of published articles, it is not uncommon for many of them, including some of the more well-known journals, to publish articles with various degrees of methodological flaws. I have provided examples of such flaws in published burn injury research.^3^

JIVR gives a high priority to publishing research reports that are in line with the needs mentioned above and which use various epidemiological study designs to address injury and violence problems. JIVR has published many articles with sufficient variability in study populations, objectives and methodologies. This can be seen in our recent issue, Vol 4: No 1. Of the seven original articles published, one used a qualitative methodology.^4^The second article used secondary data analysis on data from the World Health Organization and the United Nations data banks.^5^A retrospective cohort study design was used in the third article to examine the effect on fetal development of high doses of drugs taken as a suicide attempt during pregnancy.^6^In this issue we also published an interventional study from Sweden.^7^Ecologic study design, although of limited value in assessing the causal relationships , is a useful cost-effective study design in epidemiology. The fifth article used this methodology to investigate the association between the use of the Mental Health Act and general population suicide rates in England and Wales.^8^The last two original articles we published in Vol 4: No 1 issue of JIVR analyzed determinants of victimization from bullying,^9^and determinants of traffic injury severity.^10^
